# Alternative Applications of Trans-Oral Robotic Surgery (TORS): A Systematic Review

**DOI:** 10.3390/jcm9010201

**Published:** 2020-01-11

**Authors:** Giovanni Cammaroto, Luigi Marco Stringa, Henry Zhang, Pasquale Capaccio, Francesco Galletti, Bruno Galletti, Giuseppe Meccariello, Giannicola Iannella, Stefano Pelucchi, Ahmed Baghat, Claudio Vicini

**Affiliations:** 1 Unit of Otolaryngology, Hospital Morgagni Pierantoni, 47100 Forlì, Italy; giuseppemec@yahoo.it (G.M.); giannicola.iannella@uniroma1.it (G.I.); claudio@claudiovicini.com (C.V.); 2 Unit of Otolaryngology, University of Messina, 98100 Messina, Italy; fgalletti@unime.it (F.G.); bgalletti@unime.it (B.G.); 3 Unit of Otolaryngology, University of Ferrara, 44121 Ferrara, Italy; luigi.stringa@gmail.com (L.M.S.); stefano.pelucchi@unife.it (S.P.); 4 Unit of Otolaryngology, Head and Neck Surgery, Royal London Hospital, Whitechapel road, E1 1FR London, UK; henryzhang87@googlemail.com; 5 Department Department of biochemical, surgical and Dental sciences university of Milan, Fondazione IRCCS Ca’ Granda Ospedale Maggiore Policlinico, 20122 Milan, Italy; pasquale.capaccio@unimi.it; 6 Department of Otorhinolaryngology, Alexandria University, 21568 Alexandria, Egypt; ahmedyassinbahgat@gmail.com

**Keywords:** TORS, OSAS, benign diseases, oropharynx, larynx

## Abstract

Background: The role of robotic surgery in the field of oncology has been widely described, in particular for the tumours of the oropharynx and larynx, but its efficacy for benign pathology is inconsistent. Methods: An exhaustive review of the English literature on trans-oral robotic surgery (TORS) for benign conditions was performed using PubMed electronic database. Results: The research was performed in March 2019 and yielded more than eight hundred articles, with 103 meeting the inclusion criteria and considered in the present study. Conclusions: The application of TORS for the treatment of obstructive sleep apnoea syndrome seems to be particularly well documented. Additionally, there exists a special interest in its use where high precision in limited anatomic space is required. There are still different structural and economic limitations for the application of TORS, however, the progressive technologic innovations and the increasing adoption of robotic surgery seem to encourage the uptake of this technique.

## 1. Introduction

In recent years, robotic surgery has become a widespread technique for the treatment of different pathologies in various regions of the human body. Despite surgical field limitations, robotic surgery in the head and neck has been well established. Trans-oral robotic surgery (TORS) has its main application in the surgical treatment of cancers and among them, those of the tonsils, the base of the tongue and larynx are widely described [1]. Thanks to its advantages in accuracy and vision, interest in TORS has also been increasing for benign pathologies where aesthetic and functional impacts are relevant, avoiding in this way more invasive procedures. One of the first applications of TORS in benign pathology was described in 2005, and it was performed in a patient with a vallecular cyst [2]. With the increasing publication of data concerning the management of this technique, more centres have started to apply TORS for this kind of pathology.

The most common system used to perform TORS is the Da Vinci Surgical system, but the increasing interest in robotic surgery in the cervico-facial region has given rise to the development of alternative devices, such as Flex^®^ Robotic System, with the aim of overcoming anatomical limitations and, at the same time, improving the surgical exposure [3].

Our goal in this study is to present a systematic review of the literature regarding the use of TORS in the management of benign pathologies of the head and neck in order to demonstrate the various applications of this technique, and to better understand its role, potential and limitations.

## 2. Materials and Method 

An exhaustive review of the English literature on trans-oral robotic treatment of benign conditions was performed using PubMed electronic, EMBASE, Cochrane and CENTRAL electronic databases. Two searches were performed using the keywords (1) TORS, (2) trans-oral robotic surgery, and these words were combined with the use of the AND function to make a selective search. Each article was considered in conformance with the following inclusion criteria: (1) the use of trans-oral robotic surgery, (2) the surgical treatment of non-malignant and functional pathology in the cervico-facial region. Exclusion criteria were as follows: articles missing one or more of the abovementioned inclusion criteria and articles describing animal or cadaveric samples. A further manual search through the bibliography of the included articles was performed in some cases, trying in this way to reduce the risk of an incomplete analysis of the literature.

The Preferred Reporting Items for Systematic Reviews and Meta-Analyses (PRISMA) criteria were applied to select the papers. Secondly, the studies were stratified according to their level of evidence, using the PRISMA criteria [4]. Finally, the level of scientific evidence of the included studies was checked according to the SORT criteria (Strength of Recommendation Taxonomy) [5]. 

Data from the studies were first extracted and assessed by the principal investigator (GC), and thereafter independently analysed by the co-authors using standardized data forms.

The Quality Assessment of Studies (QUADAS-2) tool was used to evaluate relevant study design characteristics of the included studies. This type of quality assessment was designed in 2003 and updated in 2011 to help judge the risks of bias and applicability [6].

## 3. Results

The research was performed in December 2019 and yielded more than eight hundred articles, with 100 meeting the inclusion criteria and considered in the present study. 

The majority of included papers expressed a level of evidence of 3 with a strength of recommendation of C. QUADAS-2 analysis shows that there is a risk on bias when considering patient selection and the flow/timing of the studies.

**Figure 1:**
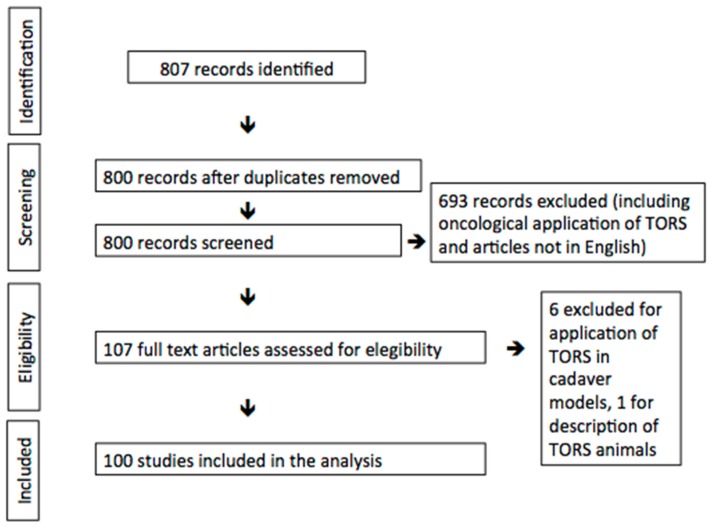
Preferred Reporting Items for Systematic Reviews and Meta-Analyses (PRISMA) graph. TORS: trans-oral robotic surgery.

In order to clearly present the different applications of TORS, we grouped each article depending on the head and neck anatomical region described or the aim of the intervention. In some cases, different anatomical regions were described in the same article, therefore we considered these works to belong to the group of the most representative region. In this way we found 1 article for oral cavity, 37 for the oropharynx, 7 for the hypopharynx, 11 for the larynx, 21 for the parapharyngeal and retropharyngeal spaces, 6 for the sublingual and submandibular glands, 5 for the thyroid space, 1 describing the trans-oral access to the sella turcica for the treatment of pituitary adenomas and 11 articles describing the use of TORS in reconstructive surgery (Table 1, Figure 1).

## 4. Discussion

Taking into account the limited quality of the existing evidence, mostly consisting of case series and observational studies, the following considerations can be made.

Despite being a relatively novel procedure, the application of TORS has increased in recent years as a consequence of advances in technology and surgical techniques. Subsequently, through the use of TORS, different anatomical regions of the head and neck can be reached in order to carry out a minimally invasive approach to the treatment of different pathologies, many of which would have traditionally been treated via more invasive, open approaches. The anatomical regions reached by TORS and described in the literature are provided below.

### 4.1. Oropharynx

The first application of robotic surgery of the head and neck was in the oropharynx, in 2005, for the removal of a vallecular cyst [2]. Indeed, the oropharynx is the most described region in the literature for TORS. Different pathologies in this sub-site have been reported to be approached by robotic surgery, and the treatment of obstructive sleep apnoea syndrome (OSAS) appears to be widespread and well documented, with several meta-analyses proving the efficacy of this procedure [7]. In particular, the robot is used to perform lingual tonsillectomy, midline glossectomy, epiglottoplasty and palatine tonsillectomy, with the aim of establishing proper air flow [7]. Not only can OSAS treatment be performed by robotic surgery, but it can also be applied in the surgical treatment of its complications, such as velopharyngeal stenosis through scar lysis and pharyngeal flaps transposition [8]. Several articles have reported the description of trans-oral robotic removal of ectopic thyroid located at the tongue base [9]. Trans-oral removal of lingual thyroglossal cyst has also been presented, offering an alternative procedure to the conventional Sistrunk technique [10]. Other conditions have been mentioned as being treated by TORS, such as a salivary duct fistula at the level of posterior tonsillar pillar [11], schwannomas and vascular lesions of the tongue base, and even the removal of a foreign body embedded in the lingual tonsil [12,13]. Since not only adults but also the paediatric population can be affected by OSAS, and as thyroglossal cyst and ectopic thyroid are congenital pathologies, TORS has potential applications in the paediatric population, despite the obvious added anatomical limitations in access [14].

### 4.2. Parapharyngeal and Retropharyngeal Spaces

As well as the oropharynx, parapharyngeal and retropharyngeal spaces seem to have caught the robotic surgeons’ eye. Several articles present the application of TORS for benign and structural pathologies of these regions. Trans-oral robotic resection of pleomorphic adenoma of the parapharyngeal space is widely described, although in a few cases this was combined with a trans-cervical approach. Other parapharyngeal masses have been reported as being treated by TORS, for example, lipomatous tumours, second branchial cysts, lymphangiomas, haemangiomas and schwannomas [15]. With regard to robotic surgery of the structural affection of this anatomical region, Eagle syndrome has been mentioned as being treated by TORS through an incision on the anterior tonsillar pillar, thereby sparing the tonsil [16]. Retropharyngeal space pathologies, such as lipomas and ectopic parathyroid glands, can be approached directly by the posterior pharyngeal wall [17]. Moreover, the retropharyngeal space can represent a way of access for the treatment of the anterior cervical spine lesions, which can be reached by the robotic arms [18].

### 4.3. Hypopharynx

Despite anatomical difficulties, the hypopharynx can be approached by robotic surgery. The piriform sinus is the most described site of benign tumours and, in this way, parathyroid adenomas, fibromas, oncocytic ductal cysts and lipomas have been reported to be removed using the TORS technique in this area [19,20,21,22]. In one case, a robotic lysis of epiglottis synechiae was performed in a patient with an unusual presentation of pemphigus vulgaris [23]. With regards to the paediatric population, TORS has been applied to the management of congenital and acquired hypopharyngeal pathologies, such as hamartomas, saccular cysts, pharyngo-esophageal strictures and lymphatic malformations [24].

### 4.4. Larynx

Technological innovations have enabled the larynx to be reached by robotic instrumentation. Robotic surgery at the level of the glottis has been described using both the da Vinci Surgical System and the more recent Flex^®^ Robotic System [25]. The latter, with its flexible system, provides an easier access to more impervious sites, allowing for better surgical exposure and tissue manipulation. Its application has been reported for the treatment of laryngeal papillomatosis, Reinke’s oedema of the vocal folds, vocal cord polyps and keratosis, and laryngeal amyloidosis [26]. Furthermore, supraglottic laryngoceles, neurofibromas and schwannomas have been approached via TORS [27,28,29]. The application of TORS in the paediatric larynx has also been described; there are reports in the literature of the robotic repair of congenital laryngeal cleft types I and II, cordectomy and arytenoidectomy for idiopathic bilateral vocal cord paralysis, and cricoid split with cartilage graft as a consequence of inhalational burns injuries [30]. Due to the tight spaces limiting movements in this region, in some cases open conversion was necessary.

### 4.5. Thyroid 

Although the traditional trans-cervical approach is still the most common approach to the thyroid space, the endeavour to minimise a visible neck scar, which has cultural implications as well as cosmetic concerns, has led to a variety of minimally invasive techniques using endoscopic or robotic technologies. Focusing on robotic application, three procedures, characterised by different access, have been described. Trans-axillary and facelift approaches are the first to have been presented. Both approaches are remote-access or “scarless”, but they require a vast amount of tissue dissection to reach the thyroid from the access point, as well as being only able to approach the ipsilateral side. As a good minimally invasive technique not only reduces the length of surgical incision, but also shortens the distance between the incision and the target, a more recent trans-oral approach has been formulated. Concerning the surgical access, the lower lip vestibule is the site of incision and, following dissection of soft tissue, the tip of the mandible is reached [31]. According to the gasless or CO_2_ insufflation technique, retractor or bariatric trocars can be positioned to allow a better visualisation of the surgical field and a wider range of motion for the robotic arms. Thanks to the robotic trans-vestibular access to the thyroid space, total thyroidectomy, hemi-thyroidectomy and parathyroidectomy can be performed in order to minimise the aesthetic impact of a surgical procedure. However, a trans-oral robotic approach to the thyroid space is not without risk; permanent paresthesia of the lower lip is the most characteristic complication described during the incision of the vestibule and the musculocutaneous flap dissection. In this way, the mental nerves can be injured, leading to transient or permanent neurological consequences. Bruising over the zygomatic region has also been described as a result of pressure from the robotic arms being applied to the patient’s face [32].

The largest case series was recently presented by Tae et al., with 79 cases. These authors compared traditional approaches with trans-oral (endoscopic or robotic) thyroid procedures, showing comparable outcomes. Robotic thyroid surgery was performed mostly to treat malignant diseases, thus underling the feasibility of this new application also for severe clinical patterns. Moreover, trans-oral procedures appeared to be quicker, while complications such as vocal cord palsy (*n* = 0 cases of permanent palsy in robotic group) and hypoparathyroidism (*n* = 1 case of permanent hypoparathyroidism) did not differ between the two techniques [33]. 

### 4.6. Sublingual and Submandibular Glands

Robotic surgery has also been described for the treatment of major salivary glands diseases, more specifically, those affecting sublingual and submandibular glands. Concerning the sublingual glands, the application of TORS for the treatment of simple or plunging ranulas has been reported, whereby complete excision of the benign lesions and the gland were carried out [34]. Additionally, salivary stones of the submandibular gland can be removed by the application of robotic instruments [35,36], and postoperative sialendoscopy demonstrated improved gland and tissue sparing, reducing symptoms and recurrence [37]. In all cases, the same complications described for the traditional trans-oral technique were noted, but the better visualisation provided by the three-dimensional monitor allowed a more precise dissection and preservation of delicate structures such as the lingual nerve and Wharton’s duct. More recently, trans-oral robotic excision of the submandibular gland has been described for the treatment of recurrent sialadenitis, providing an alternative approach to the traditional cervical approach. In the first case reported by J. Drew Prosser et al. [38], in order to safely reach the submandibular gland to allow a better dislocation of the lingual nerve, the sublingual and Wharton’s ducts were removed. Compared to the cervical approach, the trans-oral resection of the submandibular gland represents a minimally invasive alternative that avoids any risk of damage to the marginalis branch of the facial nerve during the dissection of the submandibular region, the potential recurrent disease of the remaining salivary duct, as it is removed together with the gland, and any aesthetic impacts given by a neck scar. However, even in gland removal by TORS, potential injuries of the lingual nerve, hypoglossal nerve and facial artery must be taken into account. 

### 4.7. Sella Turcica

The advent of endoscopic technology has allowed for a video-assisted trans-nasal approach to become the gold standard for pituitary adenoma surgery for more than twenty years [39]. Efforts to introduce a trans-oral approach to the pituitary have been carried out in order to reduce surgical risk. The incoming robotic technology and the continuous research of minimally invasive approaches have permitted the application of TORS for access to the sella turcica, as shown recently by Chauvet et al. [40]. Specific anatomical considerations are required to allow safe and effective access to the target site, allowing a sufficient range of motion of the robotic instrumentation. So far, all patients subjected to a trans-oral robotic surgery for pituitary adenoma excision presented a sellar type of sphenoid sinus, which represents the most favourable anatomical variation. Regarding the surgical approach, after the elevation of the palatal velum with a retractor, a nasopharyngeal mucosal incision is performed in order to expose the sphenoidal bone, and after its drilling, the pituitary fossa is reached. As shown in the first experience, the robotic sellar approach avoids any postoperative nasal discomfort or complication, but also allows inferosuperior access to the sphenoid and sella, which creates a more comfortable exposure of the adenomas. Some complications in common with traditional techniques have been reported, for example, intraoperative cavernous haemorrhage, CSF leaks and postoperative pneumocephalus in the pituitary fossa, while for the side effects specifically for TORS, transient sore throat, hypernasal speech and delayed otitis media have been reported.

### 4.8. Reconstructive Surgery

The first descriptions of TORS in reconstructive surgery were in oncological surgery, but thanks to its promising evidence, robotic surgery has generated wide interest and can be applied to the correction and reconstruction of congenial as well as iatrogenic pathologies. Post-oncological resection, robotic surgery is widely described, in particular for those tumours whose excision involves oropharyngeal tissue deficiency. Different techniques have been put forward, and some use a pedicled flap, such as the facial artery musculomucosal (FAMM) flap [41]; while others take into consideration free flaps, like those of the anterolateral thigh or radial forearm [42,43,44]. The obvious advantage of the robot is in suturing the fasciocutaneous paddle, as it allows greater manipulation and suturing in covered areas. On the contrary, time-consuming installation and the lack of haptic feedback lead many authors to still perform vascular anastomosis traditionally by the microscope. Recently, TORS has also been receiving considerable interest for its application in tissue reconstruction for congenital and iatrogenic reconstruction. In this case, robotic surgery has been described in congenital palatal and lip cleft and iatrogenic velopharyngeal stenosis correction with palatoplasty, in posterior glottic stenosis after inhalational burn injury corrected by posterior cricoid split and cartilage grafting, and for the treatment of laryngeal cleft [8,30,45].

### 4.9. Current Challenges

While there are many potential applications of TORS, it is not without potential challenges. Considerations of several of its current challenges are presented below.

- In order to evaluate the benefits and the effectiveness of trans-oral robotic surgery for the presented indications, prospective comparative studies are strongly needed.
- Currently, the high costs associated with robotic surgery represent the main limitation of its application. However, the progressive worldwide spread of this technology might reduce its high costs, resulting in its wider implementation.
- Proper training with simulator and hands-on cadaveric courses might help young surgeons to be introduced to this new surgical technique. Collecting cases in tertiary centres would probably lead to an improvement of therapeutic outcomes.
- Finally, technological innovation in robotic surgery (e.g., single-port systems) is constantly working toward reducing the complexity of the procedures and facilitating surgical handling.

## 5. Conclusions

The development and implementation of TORS is currently experiencing a period of notable progress, thanks to the continuous technological improvements and the growing interest around this new technology. Many advantages of using this technology have been recognized, such as the robotic system’s magnification and high-definition camera, which allows better visualisation of the surgical field; three-dimensionality providing better depth perception of anatomical structures and their relations; the possibility of an efficient four-handed surgery; the presence of motion scaling and tremor filtration technology, which avoids dangerous movements, allowing precise tissue dissection; and, finally, jointed instruments, which offer better access to tight and difficult areas that can be challenging to access with a traditional approach. On the other hand, many obstacles slow the propagation of TORS. Firstly, the cost of the instrumentation significantly impacts on the justification of its use in some cases, as well as the accessibility to the robotic system, as it is often shared with more than one surgical speciality [46]. Equally, the lack of dedicated equipment for head and neck surgery can hinder the progress from traditional to robotic surgery. Finally, the use of a robotic approach can be associated with the elongation of surgical times, especially with the set up and the docking phase of the surgery, although data has shown that its impact is minimal on the total operation time and that with experience it can be reduced significantly [47]. However, at the moment, robotic surgery is widely used and its application in head and neck oncologic field is widely documented and described. However, its impact on the treatment of benign tumours and functional disorders has not yet been clearly defined. In some cases, such as OSAS, TORS seems to provide an efficient therapeutic solution for both adult and for paediatric patients, whereas in other cases, further efforts and research is needed to confirm the preliminary studies, suggesting robotic technology can offer a promising alternative to traditional approaches.

## Figures and Tables

**Table 1:** Clinical data of papers included in the review.

Number of Papers	Anatomical District	Surgical Procedures	Number of Patients
1	Oral cavity	Lesion removal	1
37	Oropharynx	Base of tongue reduction; Foreign body removal; Lingual thyroid resection and cyst excision	289
3	Retropharyngeal space	Mass resection	3
2	Cervical spine	Mass resection	3
16	Parapharyngeal space	Mass resection; Styloid process resection	106
7	Hypopharynx	Mass excision;	23
11	Larynx	Laryngocele excision; Mass excision	33
5	Thyroid space	Thyroidectomy (partial or total); Parathyroidectomy	138
6	Submandibular and sublingual glands	Sialolithotomy; Lesion removal	6
1	Sella Turcica	Mass removal	4
11	Reconstructive Surgery	Reconstruction post-oropharyngectomy; Palatal cleft repair Palatoplasty; Laryngeal cleft repair	75
Tot. 100	-	-	Tot. 5835
